# *TNFAIP3*, *TNIP1*, and *MyD88* Polymorphisms Predict Septic-Shock-Related Death in Patients Who Underwent Major Surgery

**DOI:** 10.3390/jcm8030283

**Published:** 2019-02-26

**Authors:** Maria Ángeles Jiménez-Sousa, Alejandra Fadrique, Pilar Liu, Amanda Fernández-Rodríguez, Mario Lorenzo-López, Esther Gómez-Sánchez, Alicia Gómez-Sanz, María Heredia-Rodríguez, Estefanía Gómez-Pesquera, Isidoro Martínez, Eduardo Tamayo, Salvador Resino

**Affiliations:** 1 Unidad de Infección Viral e Inmunidad, Centro Nacional de Microbiología, Instituto de Salud Carlos III, 28220 Majadahonda, Spain; amandafr@isciii.es (A.F.-R.); algomez@isciii.es (A.G.-S.); imago@isciii.es (I.M.); 2 Departamento de Anestesiología y Reanimación, Hospital Clínico Universitario, 47005 Valladolid, Spain; alejandrafadrique@gmail.com (A.F.); liupili@gmail.com (P.L.); mariolorenzo17@yahoo.es (M.L.-L.); esthergzam@hotmail.com (E.G.-S.); maria_her_05@hotmail.com (M.H.-R.); egp29@hotmail.com (E.G.-P.); tamayo@med.uva.es (E.T.)

**Keywords:** *TNFAIP3*, *TNIP1*, *MyD88*, SNPs, septic shock, survival, major surgery

## Abstract

Background: In many immune-related diseases, inflammatory responses and several clinical outcomes are related to increased NF-κB activity. We aimed to evaluate whether SNPs related to the NF-κB signaling pathway are associated with higher susceptibility to infection, septic shock, and septic-shock-related death in European patients who underwent major surgery. Methods: We performed a case-control study on 184 patients with septic shock and 212 with systemic inflammatory response syndrome, and a longitudinal substudy on septic shock patients. Thirty-three SNPs within genes belonging to or regulating the NF-κB signaling pathway were genotyped by Agena Bioscience’s MassARRAY platform. Results: No significant results were found for susceptibility to infection and septic shock in the multivariate analysis after adjusting for multiple comparisons. Regarding septic-shock-related death, patients with *TNFAIP3* rs6920220 AA, *TNIP1* rs73272842 AA, *TNIP1* rs3792783 GG, and *TNIP1* rs7708392 CC genotypes had the highest risk of septic-shock-related death in the first 28 and 90 days. Also, the *MyD88* rs7744 GG genotype was associated with a higher risk of death during the first 90 days. Haplotype analysis shows us that patients with the *TNIP1* GAG haplotype (composed of rs73272842, rs3792783, and rs7708392) had a lower risk of death in the first 28 days and the *TNIP1* AGC haplotype was associated with a higher risk of death in the first 90 days. Conclusions: The SNPs in the genes *TNFAIP3*, *TNIP1,* and *MyD88* were linked to the risk of septic-shock-related death in patients who underwent major surgery.

## 1. Introduction

Sepsis is a life-threatening organ dysfunction that results from a dysregulated host response to infection [1]. Sepsis is the leading cause of admission to intensive care units (ICUs) and death in the critically ill population worldwide [2]. Septic shock is the most severe stage of sepsis and causes a substantial increase in mortality due to severe cellular and metabolic abnormalities [1]. The number of patients with sepsis and septic shock is growing worldwide, probably due to the increased number of elderly patients who suffer from more comorbidities [3,4]. The proportion of patients with sepsis who die has decreased during the last two decades [3,5], but sepsis-related mortality remains unacceptably high and constitutes a substantial cost for healthcare systems [3,6]. Identifying predictors of sepsis morbidity and mortality is a priority to provide adequate management of patients [7].

Sepsis is related to excessive inflammation that may result in a dysfunction of the immune response and tissue damage that promote organ dysfunction and even multiorgan failure [8,9]. The role of transcription factor nuclear factor-kappaB (NF-κB) in the development of organ injury and death during sepsis is widely known [10]. NF-κB also plays a central role in sepsis through its ability to modulate the expression of a large number of genes that control innate immunity, inflammation, cellular stress response, cell proliferation, and survival. As a result, activation of NF-κB promotes the development of inflammation, cell apoptosis, and endothelial damage—three key factors in the development of septic shock [11].

NF-κB is activated by many different stimuli, including microbial pathogens, cytokines, and stress, among others, through canonical or noncanonical cascades [11]. The canonical pathway is triggered by several receptors such as toll-like receptors (TLRs), NOD-like receptors (NLRs), RIG-I-like receptors (RLRs), and cytokine receptors, among others [12]. In the NF-κB signaling cascade, many proteins are involved, such as interleukin-1 receptor-associated kinases (IRAKs), toll-interacting protein (TOLLIP), and myeloid differentiation primary response 88 (MyD88), as well as microRNAs, such as the 146a (miR-146a) [13]. In quiescent cells, NF-κB is inactive due to binding to its inhibitory protein (IkB) in a protein complex located in the cytoplasm. In the septic process, immune stimuli trigger the IκB polyubiquitination and its subsequent proteasomal degradation, releasing NF-κB, which translocates to the nucleus and activates the expression of multiple target genes [11]. The regulation of the NF-κB signaling pathway is an essential step for controlling excessive immune response and tissue injury. One of the genes involved in this pathway is the tumor necrosis factor alpha-induced protein 3 (TNFAIP3), which plays a crucial role in the negative regulation of NF-κB signaling by its dual function as both a deubiquitinase and an ubiquitin ligase [12]. TNFAIP3 may collaborate with other proteins to regulate the NF-κB pathway, such as the TNFAIP3-interacting protein 1 (TNIP1) and TNF receptor-associated factor 6 (TRAF6) [13].

In patients with sepsis, growing evidence suggests that single nucleotide polymorphisms (SNPs) are critical determinants of interindividual differences both in inflammatory responses and in clinical outcomes [14]. However, there is scarce information about the role of NF-kB-signaling-pathway-related SNPs. Polymorphisms at *TLR* genes have been the most studied [14]. Furthermore, there are some previous studies that have found significant associations of SNPs at *TRAF6* [15,16], *MIR146A* [17], *IRAK-1* [18,19,20], *IRAK-4* [20,21], *MyD88* [20], and *TOLLIP* [22] genes with sepsis. Finally, several studies have demonstrated the association of both *TNFAIP3* and *TNIP1* SNPs with multiple chronic inflammatory diseases [13,23], but there has not been any study analyzing their relationship with sepsis.

In this study, we aimed to evaluate whether SNPs in several NF-κB-signaling-pathway-related genes are associated with susceptibility to infection, septic shock, and septic-shock-related death in European patients who underwent major abdominal or cardiac surgery.

## 2. Patients and Methods

### 2.1. Patients

We performed a case-control study on 396 patients who underwent major surgery (cardiac or abdominal) from the Hospital Clínico Universitario of Valladolid (Spain), between April 2008 and November 2012: (a) 184 patients who underwent major surgery and developed an infection (positive culture) and a subsequent septic shock (Septic Shock group); (b) 212 patients, with age and gender similar to the septic shock patients, who underwent major surgery and did not develop sepsis, but who did develop a systemic inflammatory response syndrome (SIRS group - control group), which is a frequent condition after major surgery. Those patients who did not have SIRS or septic shock were excluded. Furthermore, we also analyzed the survival in patients with septic shock, using two censoring points (28 and 90 days).

The study was conducted following the ethical requirements established by the Declaration of Helsinki. The Ethics Committee of Instituto de Salud Carlos III (Majadahonda) and Hospital Clínico Universitario (Valladolid) approved the study. All participants provided written informed consent. When a patient was unable to sign, the consent was signed by a family member or legal representative of the patient.

### 2.2. Clinical Data

Patients’ epidemiological and clinical data were collected from medical records. All patients underwent a major surgery, which was defined as a surgical procedure under general anesthesia and respiratory assistance. All heart surgeries involved cardiopulmonary bypass. The indication for emergency surgery included pathologies such as intestinal perforation, aortic dissection, heart disease due to stenosis of the trunk of the left coronary artery, and postoperative bleeding.

Sequential Organ Failure Assessment (SOFA score [24]) and Acute Physiology and Chronic Health Evaluation (APACHE II score [25]) for assessing the severity of sepsis were calculated within the first 24 h after septic shock diagnosis.

In this study, SIRS was considered as a clinical response to a noninfectious insult, since SIRS related to infection was excluded. The SIRS diagnosis was made during the first 24 h postsurgery. Septic shock was defined as a state of acute circulatory failure characterized by persistent arterial hypotension unexplained by other causes other than infection. Hypotension was defined by a systolic blood pressure below 90 mmHg, a mean arterial pressure <60 mmHg, or a reduction in systolic blood pressure of >40 mmHg from baseline, despite adequate volume resuscitation, in the absence of other causes for hypotension. The diagnosis of septic shock was made during the entire follow-up time postsurgery. Inotropic agents were administered early as recommended by the Surviving Sepsis Campaign: International Guidelines for Management of Sepsis and Septic Shock. All patients were treated with noradrenaline, and in some cases, adrenalin and dobutamine were also administered. Both diagnoses (SIRS and septic shock) were established according to the criteria laid down by the SCCM/ESICM/ACCP/ATS/SIS International Sepsis Definitions Conference (Sepsis-2) [26].

Antibiotic therapy for sepsis was based on our prior experience in identifying the most common bacterial pathogens associated with sepsis in our medical ICU, according to international guidelines [27]. Antibiotic administration included initial empirical treatment of methicillin-resistant *Staphylococcus aureus* with linezolid or teicoplanin and treatment of *Pseudomonas aeruginosa* with at least one of the following antibiotics: imipenem, cefepime, or piperacillin/tazobactam in association with amikacin or ciprofloxacin.

### 2.3. SNP Selection

We selected 33 SNPs via a literature search in PubMed of genes involved in the NF-κB signaling pathway and that were previously related to chronic inflammatory diseases. The selected polymorphisms are located at the following genes: *TNFAIP3*, *IRAK1*, *IRAK2*, *IRAK4*, *MIR146A*, *MyD88*, *TLR1*, *TLR4*, *TNIP1*, *TOLLIP*, and *TRAF6* (Supplemental Table S1).

### 2.4. DNA Genotyping

Total DNA from peripheral blood was extracted using the High Pure PCR Template Preparation kit (Roche Diagnostics GmbH, Mannheim, Germany). Next, DNA samples were genotyped at the Spanish National Genotyping Center (CeGen; http://www.cegen.org) by the Agena Bioscience’s MassARRAY platform (San Diego, CA, USA) using the iPLEX^®^ Gold assay design system.

### 2.5. Outcome Variables

Two main outcome variables were analyzed: (1) susceptibility to infection, septic shock, and septic-shock-related death (case-control study); (2) mortality after diagnosis of septic shock (longitudinal substudy). For survival analysis, we used two censoring points: (1) 28-day mortality (early mortality mainly related to infection [28]), which is used as the primary endpoint for severe sepsis in most clinical trials of new therapeutic approaches; (2) 90-day mortality (late mortality mainly related to causes other than sepsis [28]), which is the other primary endpoint used to evaluate excess mortality beyond the first 28 days.

### 2.6. Statistical Analysis

For the description of the study population, the differences between groups were calculated by the Mann–Whitney U test for continuous variables and the chi-squared/Fisher’s exact test for categorical variables. The NF-κB-pathway-related SNPs were analyzed for deviation from the Hardy–Weinberg equilibrium (HWE), where *p* < 0.001 was considered to be statistically significant. For SNPs in the X chromosome, HWE was calculated excluding male genotypes. 

Regarding the genetic association study, analyses were carried out for dominant, recessive, overdominant, codominant, and additive models, selecting the inheritance model that best fit our data. Firstly, in the case-control study, logistic regression was performed to investigate the association between SNPs involved in the NF-κB signaling pathway and the development of septic shock (Septic Shock group versus SIRS group). Multivariate logistic regression analysis was used to adjust for the main covariates selected by a stepwise method (forward): age, gender, smoking, drinking, comorbidities (obesity, diabetes, hypertension, chronic kidney disease, heart disease, chronic obstructive pulmonary disease (COPD), neoplasia, and liver disease), SOFA score, and type of surgery (emergency or scheduled; cardiac or abdominal). 

Secondly, in the longitudinal substudy, a survival analysis was used to evaluate mortality in the first 28 and 90 days in septic shock patients (Septic Shock group). Survival probabilities were estimated by the Kaplan–Meier product-limit method, and groups were compared using the log-rank test. In order to exclude spurious associations, multiple testing correction was carried out by the false discovery rate (FDR) with the Benjamini and Hochberg procedure. Only SNPs with a *p*-value less than 0.1 from the Kaplan–Meier method (after an FDR adjustment) were analyzed in the Cox regression analysis. All multivariate Cox regression tests were adjusted by the most significant covariates, which were selected by a stepwise method (forward), from the following list: age, gender, antibiotic treatment, peritonitis, hypertension, lactate, comorbidities (obesity, diabetes, chronic kidney disease, heart disease, COPD, neoplasia, and liver disease), SOFA score, and type of surgery (emergency or scheduled; cardiac or abdominal). Next, we analyzed the diagnostic performance of SNPs for predicting septic-shock-related death using the area under the receiver-operating characteristic (AUROC) curve. In this analysis, only the five most significant clinical variables for each time point (28 and 90 days) and the most significant SNPs resulting from the Cox regression analysis were used. The following criteria for levels of accuracy were taken into account: >0.90–1 = excellent, >0.80–0.90 = good, >0.70–0.80 = fair, and >0.60–0.70 = poor. Delong test was carried out to compare the two AUROC curves. Additionally, we analyzed the diagnostic accuracy of adding SNPs to the model by calculating sensitivity, specificity, and positive and negative predictive value. Several cut-offs were used: (a) 95% of sensitivity; (b) maximum test sensitivity plus specificity; (c) 95% of specificity.

All statistical analyses were performed using the R statistical package version 3.4.3 (R Foundation for Statistical Computing, Vienna, Austria). All *p*-values were considered significant with values of *p* < 0.05 (two-tailed). Besides, linkage disequilibrium (LD) was computed by Haploview 4.2 software, and haplotype-based association testing was performed using PLINK software. 

## 3. Results

### 3.1. Clinical Characteristics of the Study Population

Table 1 shows demographic and clinical characteristics of 396 patients who underwent cardiac or abdominal surgery and developed septic shock (*n* = 184, case group) or SIRS (*n* = 212, control group). The Septic Shock group had higher percentages of patients with chronic kidney disease, abdominal surgery, emergency surgery, and higher values of SOFA and APACHE II score, while the SIRS group had higher percentages of patients with heart disease, cancer, and cardiac surgery (*p* < 0.05). 

The baseline characteristics of the 184 septic shock patients are shown in Table 2. Overall, the median age was 73 years, 65.8% were males, and more than 50% of patients had abdominal or emergency surgeries, and infection by gram-negative bacteria. When the population was stratified by exitus versus nonexitus, the patients who died were older, had higher lactate and procalcitonin values and SOFA and APACHE II scores, more reduced period of time from surgery to septic shock diagnosis, and higher percentages of chronic kidney disease and emergency surgery (*p* < 0.05). Patients who underwent cardiac surgery or elective surgery had longer period of time from surgery to septic shock diagnosis than patients who underwent abdominal surgery or emergency surgery (Supplemental Table S2). All septic shock patients had an infection that was microbiologically confirmed. Eighty-seven percent had an adequate initial empirical treatment according to the antibiogram data.

### 3.2. Characteristics of NF-κB-Signaling-Pathway-Related SNPs

**Figure 1:**
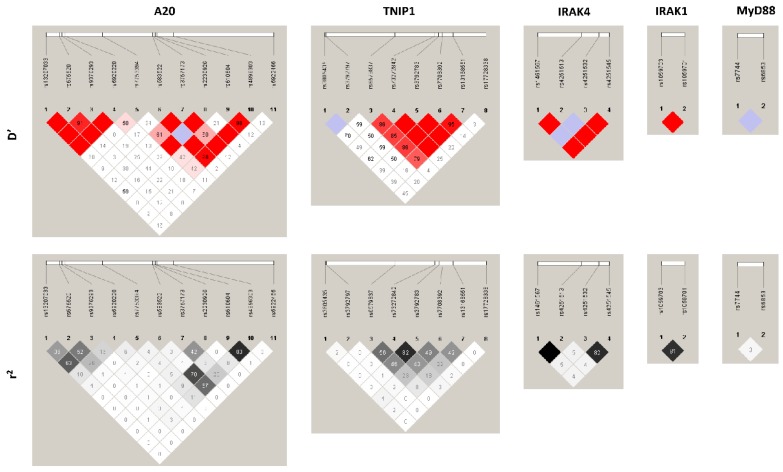
Pairwise linkage disequilibrium (LD) patterns for polymorphisms involved in the NF-κB pathway. Each diagonal represents a different SNP, with each square representing the coefficient of linkage disequilibrium (D’) or *r*^2^ data for a pairwise comparison between two SNPs.

Most of the SNPs had low/medium LD among them with a maximum of *r*^2^ = 0.83 (Figure 1). Fourteen out of 33 SNPs were located in an intronic region, nine were in an exonic region, and six in an upstream and four in the downstream region of their respective genes (Supplemental Table S1). All SNPs had a minor allelic frequency higher than 10%, except *TNFAIP3* rs2230926; *TNIP1* rs17728338, rs6579837, and rs5743867; and *TRAF6* rs16928973. Similarly, most SNPs fulfilled the HWE (*p* > 0.001), except *IRAK1* rs1059701, rs1059703; and *IRAK4* rs1461567. The genotypic frequencies were similar between the Septic Shock group and the SIRS group (Supplemental Table S1).

### 3.3. Association between NF-κB-Signaling-Pathway-Related SNPs and Susceptibility to Infection and Septic Shock

Several SNPs were associated with susceptibility to infection and septic shock, one in the univariate analysis (rs6853 at *MyD88* gene) and seven with the multivariate model (rs610604, rs6922466, rs7753394, and rs583522 at *TNFAIP3* gene; rs6579837, rs73272842, rs3792783 at *TNIP1* gene). However, none of them remained significant after adjusting for multiple comparisons (Supplemental Table S3).

### 3.4. Association between NF-κB-Signaling-Pathway-Related SNPs and Death in Septic Shock Patients

Regarding death within the first 28 days, 10 SNPs were significantly associated with death, of which only 4 remained significant after correction for multiple tests: rs6920220 in *TNFAIP3* gene (*p* = 0.007) and rs73272842, rs3792783, and rs7708392 in *TNIP1* gene (*p* = 0.025, *p* = 0.007, and *p* = 0.025, respectively). Concerning death within the first 90 days, nine SNPs showed significant associations, but only *TNFAIP3* rs6920220 (*p* = 0.007), *MyD88* rs7744 (*p* = 0.043), and both rs73272842 and rs3792783 in *TNIP1* gene (*p* = 0.033 and 0.007, respectively) remained significant after correction for multiple tests. The survival probabilities are shown in Table 3 (a full description of all 33 SNPs in Supplemental Table S4).

Table 4 shows the risk of dying in the first 28 and 90 days after a septic shock diagnosis. The *TNFAIP3* rs6920220 AA, *TNIP1* rs73272842 AA, *TNIP1* rs3792783 GG, and *TNIP1* rs7708392 CC genotypes were significantly associated with a higher death risk in the first 28 days (adjusted hazard ratio [aHR] = 8.37 [*p* = 9.57 × 10^−5^], aHR = 10.84 [*p* = 8.89 × 10^−5^], aHR = 10.06 [*p* = 2.61 × 10^−5^], and aHR = 3.58 [*p* = 0.001], respectively) and 90 days (aHR = 7.56 [*p* = 1.96 × 10^−4^], aHR = 5.68 [*p* = 0.005], aHR = 5.10 [*p* = 0.004], and aHR = 2.33 [*p* = 0.025], respectively) than *TNFAIP3* rs6920220 GG/GA, and *TNIP1* rs73272842 GG/GA, rs3792783 GA/AA, and rs7708392 GG/GC genotypes after correction for multiple tests. Moreover, *MyD88* rs7744 GG genotype was associated with a higher risk of death during the first 90 days (aHR = 4.32 [*p* = 0.030]).

Three major haplotypes for *TNIP1* SNPs (composed of rs73272842, rs3792783, and rs7708392) were also related to death in septic shock patients (Table 5). The GAG haplotype was associated with a lower risk of death in the first 28 days (aOR = 0.53 [*p* = 0.024]). The AGC haplotype was associated with a higher risk of death in the first 90 days after septic shock onset (aOR = 2.09 [*p* = 0.031]).

### 3.5. Diagnostic Performance of NF-κB-Signaling-Pathway-Related SNPs for Prediction of Septic Shock-Related Death

**Figure 2:**
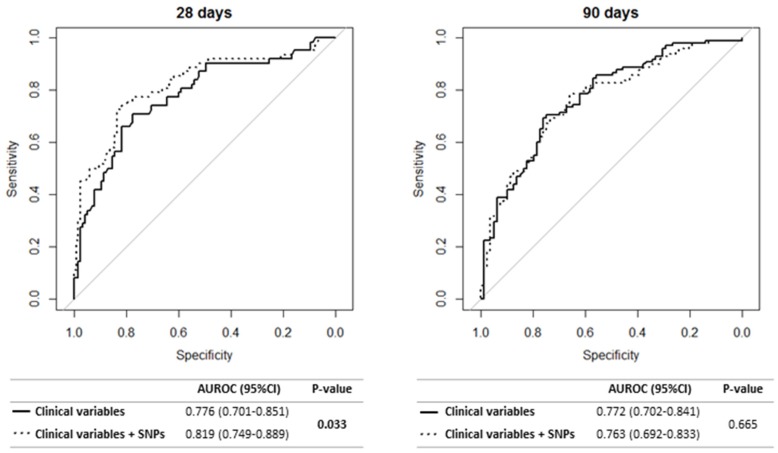
Predictive value of polymorphisms related to the NF-κB pathway in combination with clinical variables. The five most significant clinical variables for each time point were: (i) first 28 days: lactate, peritonitis, heart disease, chronic kidney disease, elective surgery; (ii) first 90 days: age, lactate, heart disease, chronic kidney disease, elective surgery. The polymorphisms that remained in both models (28 and 90 days) after stepwise were rs6920220 and rs3792783. Seven patients were excluded due to missing data for any of the covariates included in the model.

We evaluated the diagnostic accuracy for predicting septic-shock-related death from a multivariate model formed by the five most significant clinical variables and the SNPs selected in the previous analysis (MyD88 rs7744; TNFAIP3 rs6920220; and TNIP1 rs73272842, rs3792783, and rs7708392 SNPs) (Figure 2). The five most significant clinical variables for each time point were: (i) the first 28 days: lactate, peritonitis, heart disease, chronic kidney disease, elective surgery; (ii) the first 90 days: age, lactate, heart disease, chronic kidney disease, elective surgery. The SNPs that remained in both models (28 and 90 days) after stepwise selection were *TNFAIP3* rs6920220 and *TNIP1* rs3792783.

The diagnostic performance of Cox regression models with only clinical variables was higher than 0.75, both for the first 28 days and the first 90 days (AUROC = 0.776 and AUROC = 0.772, respectively). When the SNPs were added to the Cox regression models, the diagnostic performance increased significantly only for the first 28 days (AUROC = 0.819; *p* = 0.033) (Figure 2). Additionally, the model with clinical variables and SNPs had higher sensitivity and specificity values than the model including only clinical variables (Supplemental Table S5).

## 4. Discussion

In this study, we analyzed the impact of SNPs located in genes involved in the NF-κB signaling pathway on the clinical progression of septic shock. Our main findings were: (1) a possible role of eight SNPs located in *TNFAIP3*, *TNIP1*, and *MyD88* genes on susceptibility to infection and septic shock, but this association was lost after adjusting for multiple comparisons; (2) 5 of the 33 SNPs analyzed were associated with a higher risk of death in septic shock patients (*TNFAIP3* [rs6920220], *TNIP1* [rs73272842, rs3792783, rs7708392], and *MyD88* [rs7744]). To our knowledge, our study is the first description of the significant role of these five SNPs, in these three genes, in septic shock patients who underwent major surgery.

Activation of the NF-κB signaling pathway leads to the overproduction of proinflammatory cytokines and mediators, inducing inflammatory responses and tissue injury [29]. The activation of TNFAIP3 inhibits NF-κB activation, being an important suppressor of the duration and intensity of proinflammatory signaling pathways both in immune and nonimmune cells (e.g., endothelial cells) [30]. Regarding genetic background, *TNFAIP3* SNPs have been associated with numerous inflammatory and autoimmune diseases [12]. In this context, the rs6920220 SNP, located in a noncoding region upstream of *TNFAIP3*, has been linked to an increased risk of rheumatoid arthritis [31,32,33], lupus erythematosus [34], type II psoriasis [35], Sjögren’s syndrome [36], and type I diabetes [37]. However, to our knowledge, there are no previous reports investigating an association of the rs6920220 SNP with the development or prognosis of septic shock. In the current study, we found that septic shock patients carrying rs6920220 AA genotype had a seven times higher risk of death than patients with GG/GA genotype. A probable mechanism could involve the influence of the rs6920220 SNP on *TNFAIP3* expression. Ungerbäck et al. [38] described that the rs6920220 A allele was associated with lower mRNA expression of *TNFAIP3*, which reduces TNFAIP3 levels and its negative regulation of NF-κB, resulting in increased proinflammatory cytokine expression and tissue damage, which leads to a worse prognosis of the disease.

Several adaptor molecules interact with TNFAIP3, which are also involved in NF-κB inhibition. In this context, TNIP1, also known as ABIN (A20-binding inhibitor of NF-κB)-1, is a polyubiquitin-binding protein that interacts with TNFAIP3 to facilitate the binding of TNFAIP3 to polyubiquitinated mediators of NF-κB activation, and thus promotes the inhibition of NF-κB by TNFAIP3. Additionally, TNIP1 has been proposed to inhibit NF-κB activation independently of TNFAIP3, possibly by competing with NF-κB mediator proteins for polyubiquitin [39]. Several *TNIP1* SNPs have been associated with immune diseases. The *TNIP1* rs3792783 SNP, located in an intronic region, has been previously associated with antibody-positive primary Sjögren’s syndrome [36] and systemic sclerosis [40], and seems to be a risk factor for Vogt–Koyanagi–Harada syndrome [41]. Also, the *TNIP1* rs7708392 SNP, also located in an intronic region of *TNIP1,* has been related to a higher risk of developing lupus nephritis [42,43], lupus erythematosus [44,45,46], autoimmune hepatitis [47], and Sjögren’s syndrome [36]. Among septic patients, this is the first study investigating the role of *TNIP1* SNPs on this disease. Similar to the *TNFAIP3* rs6920220 SNP, *TNIP1* SNPs could have regulatory effects by conferring changes in *TNIP1* expression, possibly by being part of intronic splice enhancers or silencers. In fact, the SNP rs73272842 has shown to be the most statistically significant expression quantitative trail loci (eQTL) among several *TNIP1* SNPs in Sjogren’s syndrome patients [48]. However, as *TNIP1* SNPs may be in strong linkage disequilibrium (LD) with other regulatory SNPs, it could be a different variant that is responsible for the increased risk of death in patients with septic shock.

Homeostasis of the gastrointestinal microenvironment may be perturbed during sepsis, resulting in pathological alterations that may cause local and remote injury [49]. The tightly woven net of the intestinal epithelium may break down during sepsis, resulting in a bacterial translocation that allows pathogens and their products to access extraluminal spaces and the circulatory system. This mechanism is usually responsible for septic shock related to a bloodstream infection derived from intestinal translocation. However, the intestinal barrier disruption has also been associated with the development of the multiple organ dysfunction syndrome (MODS), independently of the type of sepsis [50]. Interestingly, it has been recently shown that TNFAIP3 and TNIP1 cooperate to preserve intestinal epithelium by preventing epithelial cell death and intestinal inflammation [45]. Therefore, it is tempting to speculate that SNPs at the *TNFAIP3* and *TNIP1* genes leading to low expression or malfunction of the corresponding proteins may be associated with the development and/or the outcome of sepsis. Supporting this hypothesis, we have found that the *TNFAIP3* AA rs6920220 genotype, which leads to low expression of TNFAIP3, was associated with an increased risk of death in patients with septic shock.

On the other hand, MyD88 is an adapter protein used by almost all TLRs to activate NF-κB. The rs7744 SNP, located at the untranslated region of *MyD88*, has been previously associated with the development of chronic inflammatory diseases, such as ulcerative colitis [51], Buerger disease [52], and coronary artery disease [53]. The function of the rs7744 SNP has not been identified yet, but it has been suggested that it could affect the binding of several microRNAs (miRNAs) [52]. Additionally, *MyD88* rs7744 SNP could affect the functionality of the noncoding circular RNA hsa-circ-0064864, which is located around this region [54] (circBase database). Circular RNAs (circRNAs) are stable RNAs that can act as miRNA sponges and decrease the number of miRNAs available for binding to their target mRNAs [55]. However, further studies would be needed to clarify their role and functionality. 

Sepsis is a complex disease, whose progression probably involves a wide number of genes and environmental factors, making it difficult to define the relative contribution of each one. In our study, we evaluated the ability to predict the death of the SNPs at genes involved in the NF-κB signaling pathway in combination with the most important clinical factors. When the *TNFAIP3*, *TNIP1,* and *MyD88* SNPs were included in the model, only the *TNFAIP3* rs6920220 and *TNIP1* rs3792783 SNPs were selected, and the predictive value significantly improved for discriminating between survivors and nonsurvivors shortly after septic shock onset (the first 28 days). Therefore, we propose that the *TNFAIP3* rs6920220 and *TNIP1* rs3792783 SNPs, besides being associated with poor survival in patients with septic shock who underwent major cardiac or abdominal surgery, could be used as prognostic markers for the early prediction of unfavorable outcomes, improving patients’ survival and quality of life. Additionally, in contrast to other biomarkers, genetic polymorphisms remain invariable throughout life, which provides important advantages concerning cost and risk to patients. Concerning surgery patients, their predictive genetic profile could be determined before the patient undergoes a surgical intervention, identifying those patients with a higher risk of a worse outcome, who could, therefore, receive more targeted attention. Additional studies would be required to elucidate the potential role of these polymorphisms in aiding clinical decision making.

### Limitations of the Study

We must clarify some limitations in order to interpret our results properly. 

Firstly, the study had a retrospective design, which may have more bias than a prospective study. In a case-control study, the choice of the control group is key to the clinical validity of the study. In this case, we selected the SIRS group as a control group because these patients were in the same conditions as the case group (undergoing major surgery), but they did not develop sepsis. Also, the selection bias due to comorbidities and the type of surgery (e.g., cardiac or abdominal surgery, or emergency or scheduled surgery) could be controlled for in part by using multivariate regression models adjusted by the most significant covariates (see Statistical analysis section). However, it should be noted that our study only included patients with major surgery of two types (abdominal and cardiac) and did not include cases of medical sepsis, something that is quite frequent in the literature.

Secondly, the limited sample size together with the low frequency of significant genotypes associated with septic-shock-related death could have limited the statistical power of this study and explained the lack of any association found for SNPs with susceptibility to infection and septic shock after FDR controlling multiple testing. Additionally, the limited sample size might have increased the rate of false positives. However, positive findings were upheld with multiple testing correction, which gives robustness to our results. 

Thirdly, the SIRS group was similar to the Septic Shock Group in age and gender, but other clinical variables were not taken into account. These differences could introduce some bias in the analysis, but in order to correct them, the logistic regression models were adjusted by the most significant clinical covariates.

Fourthly, according to Daviaud et al. [56], early deaths might be attributable to unsolvable multiorgan failure related to the primary infection, and late deaths might be related to ICU-acquired complications such as mesenteric ischemia and nosocomial infections [56]. However, we included the most significant covariates to adjust the Cox Regression analysis (see Statistical section) to get an HR value with clinical validity, although we cannot rule out that other variables not recorded in our study may have influenced the risk of death. 

## 5. Conclusions

In conclusion, *TNFAIP3*, *TNIP1*, and *MyD88* SNPs were linked to the risk of septic-shock-related death in patients who underwent major cardiac or abdominal surgery. These SNPs could be a useful tool for helping in the optimal management of patients with septic shock.

## Figures and Tables

**Table 1:** Baseline characteristics of patients with systemic inflammatory response syndrome (control group) and patients with septic shock who underwent major surgery.

Characteristics	SIRS Group	Septic Shock Group	*p*-Value *
No. patients	212	184	–
Gender (male)	136 (64.1%)	121 (65.8%)	0.819
Age (years)	72 (65–78)	73 (63–79)	0.456
Prior or pre-existing conditions			
Smoker	27 (12.7%)	33 (17.9%)	0.207
Alcoholism	6 (2.8%)	11 (6.0%)	0.203
Obesity	26 (12.3%)	29 (15.8%)	0.400
Diabetes	43 (20.3%)	25 (13.6%)	0.104
Heart disease	123 (58.0%)	83 (45.1%)	**0.012**
COPD	30 (14.1%)	32 (17.4%)	0.478
Hypertension	127 (59.9%)	102 (55.4%)	0.463
Chronic kidney disease	13 (6.1%)	29 (15.8%)	**0.004**
Cancer	76 (35.8%)	43 (23.4%)	**0.008**
Liver disease	3 (1.4%)	7 (3.8%)	0.199
Surgery			
Cardiac (versus abdominal)	117 (55.2%)	76 (41.3%)	**0.006**
Emergency (versus scheduled)	19 (9.0%)	116 (63.0%)	**<0.001**
Severity indexes			
SOFA score	3 (3–4)	9 (7–10)	**<0.001**
APACHE II score	9 (8–10)	16 (13–19)	**<0.001**

Values are expressed as median (percentile 25–percentile 75) and absolute count (percentage). * *p*-values were calculated by Chi-square test or Fisher’s exact test for categorical variables and Mann–Whitney test for continuous variables. Significant differences are shown in bold. COPD, chronic obstructive pulmonary disease; SIRS, systemic inflammatory response syndrome; SOFA, sequential organ failure assessment; APACHE, acute physiology and chronic health evaluation.

**Table 2:** Summary of epidemiological and clinical characteristics of septic shock patients who underwent major surgery.

Characteristics	All Patients	Nonexitus	Exitus	*p*-Value *
No. patients	184	80	104	–
Gender (male)	121 (65.8%)	56 (70.0%)	65 (62.5%)	0.288
Age (years)	73 (63–79)	68 (58–77)	77 (69–81)	**<0.001**
Pre-existing conditions				
Smoker	33 (17.9%)	14 (17.5%)	19 (18.3%)	0.670
Alcoholism	11 (6.0%)	4 (5.0%)	7 (6.7%)	0.597
Obesity	29 (15.8%)	12 (15.0%)	17 (16.3%)	0.654
Diabetes	25 (13.6%)	11 (13.8%)	14 (13.5%)	0.679
Heart disease	83 (45.1%)	33 (41.3%)	50 (48.1%)	0.419
COPD	32 (17.4%)	14 (17.5%)	18 (17.3%)	0.679
Hypertension	102 (55.4%)	43 (53.8%)	59 (56.7%)	0.606
Chronic kidney disease	29 (15.8%)	4 (5.0%)	25 (24.0%)	**0.001**
Cancer	43 (23.4%)	14 (17.5%)	29 (27.9%)	0.099
Liver disease	7 (3.8%)	3 (3.8%)	4 (3.8%)	0.679
Surgery				
Cardiac (versus abdominal)	76 (41.3%)	38 (47.5%)	38 (36.5%)	0.134
Emergency (versus scheduled)	116 (63.0%)	40 (50.0%)	28 (73.1%)	0.001
Severity				
Time to septic shock (days)	1(0–4)	2 (1–5)	1 (0–4)	**0.047**
Late septic shock (>4 days)	41 (22.3%)	20 (25.0%)	21 (20.2%)	0.437
Lactate (mg/dL)	26.0 (16.0–42.2)	21.0 (14.0–30.0)	30.0 (18.0–49.0)	**<0.001**
Lactate (>18 mg/dL or 2 mmol/L)	122 (66.3%)	45 (56.3%)	77 (74%)	**0.040**
White Blood Cell (×10^3^ cells/mm^3^)	14.9 (9.4–20.3)	16.1 (10.5–20.6)	13.7 (9.1–20.3)	0.358
C-Reactive protein (mg/L)	241.0 (130.2–307.2)	236.4 (123.8–307.7)	241.5 (133.5–306.9)	0.951
Procalcitonin (ng/mL)	5.0 (1.7–20.2)	3.5 (1.2–17.2)	5.9 (2.0–32.8)	**0.033**
SOFA score	9 (7–10)	8 (7–10)	9 (8–11)	**0.004**
APACHE II score	16 (13–19)	15 (12–18)	18 (14–21)	**<0.001**
Exitus				
At 7 days	23 (12.5%)	–	23 (22.1%)	–
At 28 days	66 (35.9%)	–	66 (63.5%)	–
At 90 days	102 (55.4%)	–	102 (98.1%)	–
Microorganism isolated				
Gram-positive	94 (51.1%)	40 (50.0%)	54 (51.9%)	0.796
Gram-negative	98 (53.3%)	47 (58.8%)	51 (49.0%)	0.191
Fungus	38 (20.7%)	13 (16.3%)	25 (24.0%)	0.196
Site of infection				
Catheter bacteraemia	66 (35.9%)	35 (43.8%)	31 (29.8%)	0.051
Surgical site infection	47 (25.5%)	20 (25.0%)	27 (26.0%)	0.882
Urinary tract infection	19 (10.3%)	8 (10.0%)	11 (10.6%)	0.899
Endocarditis	10 (5.4%)	4 (5.0%)	6 (5.8%)	0.820
Peritonitis	83 (45.1%)	30 (37.5%)	53 (51.0%)	0.069
Pneumonia	90 (48.9%)	42 (52.5%)	48 (46.2%)	0.393
Adequate initial empirical treatment	161 (87.5%)	69 (86.3%)	92 (88.5%)	0.653

Values are expressed as median (percentile 25–percentile 75) and absolute count (percentage). * *p*-values were calculated by Chi-square test or Fisher’s exact test for categorical variables and Mann–Whitney test for continuous variables. Significant differences are shown in bold. Note that patients may have had more than one organism cultured. COPD, chronic obstructive pulmonary disease; SOFA, sequential organ failure assessment; APACHE, acute physiology and chronic health evaluation.

**Table 3:** Survival probabilities at 28 and 90 days (Kaplan–Meier product-limit method) for SNPs related to the NF-κB signaling pathway in septic shock patients who underwent major abdominal or cardiac surgery.

Gene	SNPs	Genotype	*n*	28 Days			90 Days		
				Deaths	*p* *	*p* **	Deaths	*p* *	*p* **
*MyD88*	rs7744	AA/AG	178	63 (35.4%)	**0.026**	0.123	98 (55.1%)	**0.005**	**0.043**
		GG	4	3 (75.0%)			4 (100%)		
*TNFAIP3*	rs6920220	GG/GA	176	61 (34.7%)	**2.28 × 10** **^−^** **^4^**	**0.007**	97 (55.1%)	**2.28 × 10** **^−^** **^4^**	**0.007**
		AA	7	5 (71.4%)			5 (71.4%)		
*TNIP1*	rs73272842	GG/GA	178	62 (34.8%)	**0.003**	**0.025**	98 (55.1%)	**0.003**	**0.033**
		AA	5	4 (80.0%)			4 (80%)		
	rs3792783	AA/AG	177	61 (34.5%)	**4.40 × 10** **^−^** **^4^**	**0.007**	97 (54.8%)	**4.40 × 10** **^−^** **^4^**	**0.007**
		GG	6	5 (83.3%)			5 (83.3%)		
	rs7708392	GG/GC	169	57 (33.7%)	**0.003**	**0.025**	92 (54.4%)	**0.013**	**0.083**
		CC	14	9 (64.3%)			10 (71.4%)		

Values are expressed as absolute count and percentage. * *p*-values were calculated by log-rank tests; ** *p*-values were corrected for multiple testing using the false discovery rate (FDR) with Benjamini and Hochberg procedure. Significant differences are shown in bold. SNPs, single nucleotide polymorphisms; NF-κB, nuclear factor kappa-light-chain-enhancer of activated B cells; TNFAIP3, TNF alpha-induced protein 3; MyD88, innate immune signal transduction adaptor; TNIP1, TNFAIP3 interacting protein 1.

**Table 4:** Risk of death in septic shock patients who underwent major cardiac or abdominal surgery according to SNPs located in genes related to the NF-κB signaling pathway.

Genes	SNPs	Day 28			Day 90		
		aHR (95% CI)	*p* *	*p* **	aHR (95% CI)	*p* *	*p* **
*MyD88*	rs7744 (GG)	1.64 (0.36; 7.56)	0.525	0.525	4.32 (1.15; 16.23)	**0.030**	**0.030**
*TNFAIP3*	rs6920220 (AA)	8.37 (2.97; 23.55)	**5.74** **× 10** **^−^** **^5^**	**9.57** **× 10** **^−^** **^5^**	7.56 (2.88; 19.84)	**3.92** **× 10** **^−^** **^5^**	**1.96** **× 10** **^−^** **^4^**
*TNIP1*	rs73272842 (AA)	10.84 (3.50; 33.55)	**3.56** **× 10** **^−^** **^5^**	**8.89** **× 10** **^−^** **^5^**	5.68 (1.79; 18.06)	**0.003**	**0.005**
	rs3792783 (GG)	10.06 (3.73; 27.17)	**5.22** **× 10** **^−^** **^6^**	**2.61** **× 10** **^−^** **^5^**	5.10 (1.87; 13.87)	**0.001**	**0.004**
	rs7708392 (CC)	3.58 (1.66; 7.72)	**0.001**	**0.001**	2.33 (1.14; 4.76)	**0.020**	**0.025**

Values are expressed as hazard ratio and 95% confidence interval. * *p*-values were calculated by Cox regression tests; ** *p*-values were corrected for multiple testing using the false discovery rate (FDR) with Benjamini and Hochberg procedure. Statistically significant differences are shown in bold. aHR, adjusted hazard ratio; 95% CI, 95% confidence interval; SNPs, single nucleotide polymorphisms; NF-κB, nuclear factor kappa-light-chain-enhancer of activated B cells; TNFAIP3, TNF alpha-induced protein 3; MyD88, innate immune signal transduction adaptor; TNIP1, TNFAIP3 interacting protein 1.

**Table 5:** Association between *TNIP1* haplotypes and death in septic shock patients.

	*TNIP1* Haplotypes			Association		
Exitus	rs73272842	rs3792783	rs7708392	Freq.	aOR (95% CI)	*p*-Value
28 days	G	A	G	0.745	0.53 (0.30; 0.92)	**0.024**
	A	G	C	0.122	1.90 (0.96; 3.79)	0.069
	G	A	C	0.111	1.59 (0.75; 3.37)	0.227
90 days	G	A	G	0.745	0.62 (0.36; 1.05)	0.071
	A	G	C	0.122	2.09 (1.05; 4.17)	**0.031**
	G	A	C	0.111	1.08 (0.53; 2.20)	0.840

Values are expressed as odds ratio and 95% confidence interval. *p*-values were calculated by multivariate logistic regression adjusted by the most important clinical and epidemiological characteristics. Only haplotypes with frequency >0.1 are shown. Significant differences are shown in bold. aOR adjusted odds ratio; 95% CI, 95% confidence interval; SNPs, single nucleotide polymorphisms; NF-κB, nuclear factor kappa-light-chain-enhancer of activated B cells; TNIP1, TNFAIP3 (TNF alpha-induced protein 3) interacting protein 1.

## Supplementary Materials

The following are available online at https://www.mdpi.com/2077-0383/8/3/283/s1, Table S1: Genotypic frequencies of SNPs related to the NF-κB signaling pathway, Table S2: Description of the period of time from surgery to septic shock diagnosis stratifying by the type of surgery, Table S3: Association between SNPs related to the NF-kB signaling pathway and susceptibility to infection and septic shock, Table S4: Survival probabilities at 28 and 90 days (Kaplan-Meier product-limit method) for SNPs related to the NF-kB signaling pathway in septic shock patients who underwent major abdominal or cardiac surgery, Table S5: Diagnostic accuracy of the predictive models of polymorphisms related to the NF-κB pathway in combination with clinical variables.
