# Supplementary material for: TNFAIP3, TNIP1, and MyD88 Polymorphisms Predict Septic-Shock-Related Death in Patients Who Underwent Major Surgery
Source: J Clin Med. 2019 Feb 26;8(3):283. doi: 10.3390/jcm8030283 (PMC6463255; doi:10.3390/jcm8030283)
Supplement: Supplementary file 1 [file jcm-08-00283-s001.pdf]

Supplemental Table S1. Genotypic frequencies of SNPs related to the NF-kB signaling pathway.

Gene	SNP	Chr Pos	Location	SIRS			HWE	Septic shock			HWE	p-value
TNFAIP3	rs2230926	6:137874929	Exon 3/9	TT, 90.6%	TG, 9.0%	GG, 0.5%	0.598	TT, 91.8%	TG, 7.1%	GG, 1.1%	0.007	0.596
	rs6920220	6:137685367	Upstream	GG, 67.8%	GA, 30.8%	AA, 1.4%	0.309	GG, 66.8%	GA, 29.3%	AA, 3.8%	0.725	0.351
	rs13207033	6:137644281	Upstream	GG, 44.3%	GA, 47.2%	AA, 8.5%	0.408	GG, 46.7%	GA, 43.5%	AA, 9.8%	0.923	0.740
	rs610604	6:137878280	Intron 6/8	TT, 48.6%	TG, 41.5%	GG, 9.9%	0.812	TT, 44.0%	TG, 47.3%	GG, 8.7%	0.275	0.512
	rs6922466	6:138123793	Downstream	AA, 54.7%	AG, 38.2%	GG, 7.1%	0.903	AA, 62.3%	AG, 31.7%	GG, 6.0%	0.329	0.313
	rs7753394	6:137764111	Upstream	TT, 32.2%	TC, 50.2%	CC, 17.5%	0.785	TT, 27.9%	TC, 49.2%	CC, 22.9%	0,850	0.355
	rs675520	6:137672095	Upstream	GG, 27.4%	GA, 52.4%	AA, 20.3%	0.601	GG, 29.3%	GA, 49.5%	AA, 21.2%	0.954	0.844
	rs9376293	6:137678149	Upstream	TT, 28.3%	TC, 56.6%	CC, 15.1%	0.128	TT, 32.1%	TC, 51.6%	CC, 16.3%	0.424	0.605
	rs4896303	6:137912948	Downstream	GG, 44.3%	GC, 42.5%	CC, 13.2%	0.555	GG, 41.3%	GC, 45.7%	CC, 13.0%	0.917	0.801
	rs3757173	6:137869017	Intron 1/8	AA, 80.2%	AG, 17.5%	GG, 2.4%	0.242	AA, 82.6%	AG, 14.1%	GG, 3.3%	0.001	0.594
	rs583522	6:137868747	Intron 1/8	TT, 59.3%	TC, 35.4%	CC, 5.3%	0.995	TT, 54.4%	TC, 41.2%	CC, 4.4%	0.182	0.490
IRAK1	rs1059701	X:154019032	Exon 4/14	AA, 76.4%	AG, 8.0%	GG, 15.6%	0.004	AA, 79.8%	AG, 6.5%	GG, 13.7%	**<0.001**	0.715
	rs1059703	X:154013378	Exon 12/14	AA, 78.8%	AG, 8.0%	GG, 13.2%	0.011	AA, 83.2%	AG, 5.4%	GG, 11.4%	**<0.001**	0.483
IRAK2	rs708035	3:10234479	Exon 11/13	AA, 37.3%	AT, 44.8%	TT, 17.9%	0.490	AA, 39.3%	AT, 45.4%	TT, 15.3%	0.614	0.770
IRAK4	rs4251532	12:43784280	Intron 9/10	CC, 74.9%	CT, 22.7%	TT, 2.4%	0.667	CC, 73.4%	CT, 24.5%	TT, 2.2%	0.913	0.927
	rs4251513	12:43780401	Intron 7/10	CC, 25.0%	CG, 52.8%	GG, 22.2%	0.570	CC, 28.8%	CG, 53.3%	GG, 17.9%	0.290	0.494
	rs1461567	12:43770886	Intron 2/10	GG, 53.8%	GA, 46.2%	AA, 0.0%	0.003	GG, 49.5%	GA, 50.5%	AA, 0.0%	**<0.001**	0.449
	rs4251545	12:43786492	Exon 10/11	GG, 75.9%	GA, 22.6%	AA, 1.4%	0.847	GG, 77.2%	GA, 21.2%	AA, 1.6%	0.864	0.940
miR-146a	rs2910164	5:160485411	Exon 1/1	GG, 57.1%	GC, 36.3%	CC, 6.6%	0.799	GG, 53.1%	GC, 40.4%	CC, 6.5%	0.673	0.694
MyD88	rs6853	3:38142879	Exon 5/5	AA, 74.1%	AG, 24.1%	GG, 1.9%	0.971	AA, 65.8%	AG, 33.2%	GG, 1.1%	0.058	0.104
	rs7744	3:38142530	Exon 5/5	AA, 72.6%	AG, 25.0%	GG, 2.4%	0.887	AA, 76.5%	AG, 21.3%	GG, 2.2%	0.516	0.652
TLR1	rs5743618	4:38797027	Exon 4/4	AA, 20.3%	AC, 51.9%	CC, 27.8%	0.661	AA, 24.0%	AC, 52.0%	CC, 24.0%	0.605	0.557
TLR4	rs1927911	9:117707776	Intron 1/2	GG, 55.7%	GA, 44.3%	AA, 0.0%	0.004	GG, 59.0%	GA, 41.0%	AA, 0.0%	0.010	0.568
TNIP1	rs13168551	5:151083077	Intron 1/17	TT, 33.5%	TC, 49.5%	CC, 17.0%	0.859	TT, 34.2%	TC, 46.2%	CC, 19.6%	0.449	0.739
	rs7708392	5:151077924	Intron 1/17	GG, 59.0%	GC, 36.2%	CC, 4.8%	0.801	GG, 56.5%	GC, 35.9%	CC, 7.6%	0.439	0.493
	rs3805435	5:151021735	Downstream	TT, 84.0%	TC, 16.0%	CC, 0.0%	0.385	TT, 81.5%	TC, 17.4%	CC,1.1%	0.842	0.356
	rs3792797	5:151022330	Downstream	CC, 61.8%	CA, 32.5%	AA, 5.7%	0.607	CC, 65.2%	CA, 31.0%	AA, 1.1%	0.943	0.617
	rs17728338	5:151098757	Upstream	GG, 89.6%	GA, 10.4%	AA, 0.0%	0.583	GG, 86.4%	GA, 13.6%	AA, 0.0%	0.466	0.407
	rs6579837	5:151055333	Intron 6/17	GG, 77.6%	GT, 21.9%	TT, 0.5%	0.424	GG, 82.1%	GT, 16.8%	TT, 1.1%	0.774	0.403
	rs73272842	5:151074327	Intron 1/17	GG, 74.5%	GA, 24.1%	AA, 1.4%	0.725	GG, 78.3%	GA, 19.0%	AA, 2.7%	0.122	0.351
	rs3792783	5:151076171	Intron 1/17	AA, 71.7%	AG, 26.4%	GG, 1.9%	0.767	AA, 74.5%	AG, 22.3%	GG, 3.3%	0.192	0.446

TOLLIP	rs5743867	11:1307121	Intron 1/5	AA, 76.4%	AG, 21.7%	GG, 1.9%	0.805	AA, 81.5%	AG, 17.9%	GG, 0.5%	0.569	0.326
TRAF6	rs16928973	11:36492695	Intron 6/7	CC, 96.2%	CT, 3.8%	TT, 0.0%	0.846	CC, 97.3%	CT, 2.7%	TT, 0.0%	0.852	0.760

Statistics: Values are expressed as absolute count and percentage. P-values were calculated by Chi-square test or Fisher's exact test. Significant differences are shown in bold.

Abbreviations: SNPs, single nucleotide polymorphisms; NF-kB, nuclear factor kappa-light-chain-enhancer of activated B cells; HWE, Hardy Weinberg equilibrium; p-value, level of significance; TNFAIP3, TNF alpha induced protein 3; IRAK1, interleukin 1 receptor associated kinase 1; IRAK2, interleukin 1 receptor associated kinase 2; IRAK4, interleukin 1 receptor associated kinase 4; miR, microRNA; MyD88, innate immune signal transduction adaptor; TLR1, toll like receptor 1; TLR4, toll like receptor 4; TNIP1, TNFAIP3 interacting protein 1; TOLLIP, toll interacting protein; TRAF6, TNF receptor associated factor 6.

Supplemental Table S2. Description of the period of time from surgery to septic shock diagnosis stratifying by the type of surgery.

Characteristics	All Patients	Cardiac	Abdominal	p-value	Elective	Emergency	p-value
Time to septic shock (days)	1(0-4)	2 (1-6)	1 (0-3)	**<0.001**	**3 (1-6)**	**1 (0-2)**	**<0.001**
Late septic shock (> 4 days)	41 (22.3%)	22 (28.9%)	19 (17.6%)	0.068	22 (32.4%)	19 (16.4%)	**0.012**

Statistics: Values are expressed as median (percentile 25-percentile 75) and absolute count (percentage). (*), P-values were calculated by Chi-square test for categorical variables and Mann-Whitney test for continuous variables. Significant differences are shown in bold.

Supplemental Table S3. Association between SNPs related to the NF-kB signaling pathway and susceptibility to infection and septic shock.

			Univariate		Multivariate		
**Gene**	**SNP**	**Model**	**OR (95%CI)**	**p-value**	**aOR (95%CI)**	**p-value ***	**p-value ****
**TNFAIP3**	rs2230926	overdominant	0.77 (0.37-1.61)	0.491	1.79 (0.09-33.49)	0.694	0.765
	rs6920220	dominant	1.04 (0.68-1.58)	0.845	0.82 (0.25-2.67)	0.752	0.775
	rs13207033	overdominant	0.86 (0.57-1.28)	0.462	2.66 (0.78-8.97)	0.115	0.263
	rs610604	overdominant	1.26 (0.84-1.88)	0.249	3.68 (1.01-13.40)	**0.048**	0.225
	rs6922466	dominant	0.73 (0.48-1.09)	0.128	0.15 (0.03-0.63)	**0.009**	0.091
	rs7753394	overdominant	0.95 (0.64-1.42)	0.834	6.59 (1.66-26.15)	**0.007**	0.091
	rs675520	additive	0.97 (0.73-1.29)	0.878	0.59 (0.26-1.32)	0.203	0.372
	rs9376293	additive	0.94 (0.69-1.27)	0.701	1.46 (0.61-3.53)	0.391	0.615
	rs4896303	dominant	1.13 (0.75-1.68)	0.543	3.06 (0.87-10.72)	0.080	0.263
	rs3757173	overdominant	0.77 (0.45-1.34)	0.368	1.41 (0.18-10.54)	0.735	0.775
	rs583522	overdominant	1.27 (0.84-1.92)	0.239	4.61 (1.19-17.72)	**0.026**	0.172
**IRAK1**	rs1059701	additive	0.91 (0.61-1.35)	0.642	1.51 (0.48-4.74)	0.477	0.642
	rs1059703	additive	0.85 (0.56-1.30)	0.470	1.52 (0.45-5.07)	0.492	0.642
**IRAK2**	rs708035	additive	0.91 (0.68-1.20)	0.511	0.49 (0.20-1.16)	0.108	0.263
**IRAK4**	rs4251532	recessive	0.91 (0.24-3.46)	0.897	0.28 (0.01-8.17)	0.461	0.642
	rs4251513	overdominant	1.01(0.68-1.51)	0.932	2.69 (0.81-8.90)	0.103	0.263
	rs1461567	dominant	1.18 (0.80-1.76)	0.391	1.40 (0.42-4.63)	0.576	0.704
	rs4251545	additive	0.95 (0.62-1.45)	0.829	0.56 (0.17-1.79)	0.330	0.573
**miR-146a**	rs2910164	overdominant	1.19 (0.79-1.78)	0.401	2.93 (0.75-11.38)	0.120	0.263
**MyD88**	rs6853	overdominant	1.56 (1.01-2.43)	**0.046**	2.54 (0.68-9.41)	0.163	0.326
	rs7744	recessive	0.92 (0.24-3.49)	0.909	0.02 (0.01-92.89)	0.386	0.615
**TLR1**	rs5743618	dominant	1.21 (0.77-1.91)	0.393	0.55 (0.13-2.26)	0.412	0.619
**TLR4**	rs1927911	dominant	0.87 (0.58-1.30)	0.502	0.43 (0.13-1.42)	0.168	0.326
**TNIP1**	rs13168551	overdominant	0.87 (0.58-1.29)	0.508	0.29 (0.08-1.08)	0.066	0.242
	rs7708392	recessive	1.64 (0.71-3.80)	0.243	6.49 (0.66-63.74)	0.108	0.263
	rs3805435	additive	1.25 (0.76-2.06)	0.376	1.32 (0.32-5.41)	0.696	0.765
	rs3792797	additive	0.85 (0.60-1.20)	0.368	0.72 (0.27-1.87)	0.506	0.642
	rs17728338	dominant	1.35 (0.73-2.50)	0.326	6.07 (0.93-39.56)	0.059	0.242

	rs6579837	additive	0.80 (0.50-1.29)	0.373	0.14 (0.03-0.62)	**0.010**	0.091
	rs73272842	overdominant	0.74 (0.45-1.20)	0.226	0.17 (0.03-0.85)	**0.031**	0.172
	rs3792783	overdominant	0.79 (0.50-1.26)	0.341	0.13 (0.03-0.63)	**0.011**	0.091
**TOLLIP**	rs5743867	additive	0.71 (0.45-1.13)	0.154	0.75 (0.19-2.87)	0.682	0.765
**TRAF6**	rs16928973	dominant	0.71 (0.22-2.21)	0.558	0.71 (0.01-32.51)	0.865	0.865

Statistics: Values are expressed as odds ratio and 95% confidence interval. (*), p-values were calculated by logistic regression analysis. (**), p-values were corrected for multiple testing using the false discovery rate (FDR) with Benjamini and Hochberg procedure. Multivariate models were adjusted by the most significant clinical and epidemiological characteristics (see Statistical analysis section). Statistically significant differences are shown in bold.

Abbreviations: OR, odds ratio; aOR, adjusted odds ratio; 95%CI, 95% of confidence interval; p-value, level of significance; SNPs, single nucleotide polymorphisms; NF-kB, nuclear factor kappa-light-chain-enhancer of activated B cells; TNFAIP3, TNF alpha induced protein 3; IRAK1, interleukin 1 receptor associated kinase 1; IRAK2, interleukin 1 receptor associated kinase 2; IRAK4, interleukin 1 receptor associated kinase 4; miR, microRNA; MyD88, innate immune signal transduction adaptor; TLR1, toll like receptor 1; TLR4, toll like receptor 4; TNIP1, TNFAIP3 interacting protein 1; TOLLIP, toll interacting protein; TRAF6, TNF receptor associated factor 6.

Supplemental Table S4. Survival probabilities at 28 and 90 days (Kaplan-Meier product-limit method) for SNPs related to the NF-kB signaling pathway in septic shock patients who underwent major abdominal or cardiac surgery.

				28 days				90 days			
**Gene**	**SNP**	**Model**	**Geno**	**N**	**Deaths**	**p-value ***	**p-value ****	**N**	**Deaths**	**p-value ***	**p-value ****
**TNFAIP3**	rs2230926	res	TT+GT	181	66 (36.5)	0.348	0.638	181	101 (55.8)	0.801	0.812
			GG	2	0(0)			2	1 (50)		
	rs6920220	res	GG+GA	176	61 (34.7)	**<0.001**	**0.007**	176	97 (55.1)	**<0.001**	**0.007**
			AA	7	5 (71.4)			7	5 (71.4)		
	rs13207033	res	GG+GA	165	56 (33.9)	0.055	0.182	165	91 (55.2)	0.129	0.305
			AA	18	10 (55.6)			18	11 (61.1)		
	rs610604	res	TT+TG	167	58 (34.7)	0.449	0.701	167	90 (53.9)	0.628	0.743
			GG	16	8 (50.0)			16	12 (75.0)		
	rs6922466	dom	AA	114	38 (33.3)	0.243	0.501	114	61 (53.5)	0.105	0.277
			GG+GA	68	28 (41.2)			68	41 (60.3)		
	rs7753394	dom	TT	50	23 (46.0)	0.130	0.306	50	37 (74.0)	0.067	0.202
			TC+CC	132	43 (32.6)			132	65 (49.2)		
	rs675520	res	GG+GA	144	51 (35.4)	0.594	0.726	144	79 (54.9)	0.732	0.780
			AA	39	15 (38.5)			39	23 (59.0)		
	rs9376293	res	TT+TC	153	54 (35.3)	0.685	0.766	153	85 (55.6)	0.379	0.549
			CC	30	12 (40.0)			30	17 (56.7)		
	rs4896303	res	GG+GC	159	55 (34.6)	0.531	0.701	159	86 (54.1)	0.812	0.812
			CC	24	11 (45.8)			24	16 (66.7)		
	rs3757173	res	AA+AG	177	66 (37.3)	0.084	0.213	177	100 (56.5)	0.251	0.453
			GG	6	0 (0)			6	2 (33.3)		
	rs583522	res	TT+TC	173	59 (34.1)	**0.041**	0.160	173	93 (53.8)	**0.038**	0.172
			CC	8	6 (75.0)			8	8 (100)		
**IRAK1**	rs1059701	addA	GG+G	25	7 (28.0)	0.554	0.703	25	11 (44.0)	0.369	0.549
			GA+A	113	41 (36.3)			113	63 (55.8)		
			AA	44	18 (40.9)			44	28 (63.6)		
	rs1059703	addA	GG+G	21	5 (23.8)	0.401	0.696	21	8 (38.1)	0.245	0.453
			GA+A	115	41 (35.7)			115	64 (55.7)		

			AA	47	20 (42.6)			47	30 (63.8)		
**IRAK2**	rs708035	res	AA+AT	155	56 (36.1)	0.818	0.843	155	86 (55.5)	0.593	0.743
			TT	27	10 (37.0)			27	16 (59.3)		
**IRAK4**	rs4251532	res	CC+CT	179	65 (36.3)	0.733	0.781	179	101 (56.4)	0.688	0.756
			TT	4	1 (25.0)			4	1 (25.0)		
	rs4251513	over	CC+GG	86	34 (39.5)	0.344	0.638	86	51 (59.3)	0.399	0.549
			CG	97	32 (33.0)			97	51 (52.6)		
	rs1461567	dom	GG	90	28 (31.1)	0.231	0.501	90	41 (45.6)	0.144	0.316
			AG	93	38 (40.9)			93	61 (65.6)		
	rs4251545	over	AA+GG	144	53 (36.8)	0.697	0.766	144	82 (56.9)	0.398	0.549
			AG	39	13 (33.3)			39	20 (51.3)		
**miR-146a**	rs2910164	over	CC+GG	108	39 (36.1)	0.528	0.701	108	61 (56.5)	0.261	0.453
			CG	74	27 (36.5)			74	40 (54.1)		
**MyD88**	rs6853	over	AA+GG	123	50 (40.7)	0.084	0.213	123	79 (64.2)	**0.047**	0.172
			AG	60	16 (26.7)			60	23 (38.3)		
	rs7744	res	AA+AG	178	63 (35.4)	**0.026**	0.123	178	98 (55.1)	**0.005**	**0.043**
			GG	4	3 (75.0)			4	4 (100.0)		
**TLR1**	rs5743618	over	CC+AA	87	39 (44.8)	**0.022**	0.123	87	55 (63.2)	**0.040**	0.172
			AC	95	26 (27.4)			95	46 (48.4)		
**TLR4**	rs1927911	dom	GG	108	39 (36.1)	0.901	0.901	108	63 (58.3)	0.614	0.743
			AG	74	27 (36.5)			74	39 (52.7)		
**TNIP1**	rs13168551	over	TT+CC	98	42 (42.9)	**0.066**	0.199	98	61 (62.2)	0.109	0.277
			TC	85	24 (28.2)			85	41 (48.2)		
	rs7708392	res	GG+GC	169	57 (33.7)	**0.003**	**0.025**	169	92 (54.4)	**0.013**	0.083
			CC	14	9 (64.3)			14	10 (71.4)		
	rs3805435	res	TT+TC	181	65 (35.9)	0.631	0.743	181	101 (55.8)	0.631	0.743
			CC	2	1 (50.0)			2	1 (50.0)		
	rs3792797	over	CC+AA	127	53 (41.7)	**0.023**	0.123	127	74 (58.3)	0.295	0.487
			CA	56	13 (23.2)			56	28 (50.0)		
	rs17728338	dom	GG	159	56 (35.2)	0.483	0.701	159	85 (53.5)	0.054	0.177
			AG	24	10 (41.7)			24	17 (70.8)		
	rs6579837	res	GG+GT	181	64 (35.4)	**0.044**	0.160	181	100 (55.2)	**0.044**	0.172

			TT	2	2 (100)			2	2 (100.0)		
	rs73272842	res	GG+GA	178	62 (34.8)	**0.003**	**0.025**	178	98 (55.1)	**0.003**	**0.033**
			AA	5	4 (80.0)			5	4 (80.0)		
	rs3792783	res	AA+AG	177	61 (34.5)	**<0.001**	**0.007**	177	97 (54.8)	**<0.001**	**0.007**
			GG	6	5 (83.3)			6	5 (83.3)		
**TOLLIP**	rs5743867	over	AA+GG	150	53 (35.3)	0.521	0.701	150	87 (58.0)	0.660	0.751
			AG	33	13 (39.4)			33	15 (45.5)		
**TRAF6**	rs16928973	dom	CC	178	65 (36.5)	0.477	0.701	178	100 (56.2)	0.259	0.453
			CT	5	1 (20.0)			5	2 (40.0)		

Statistics: Values are expressed as absolute count and percentage. (*), p-values were calculated by log-rank tests; (**), p-values were corrected for multiple testing using the false discovery rate (FDR) with Benjamini and Hochberg procedure. Significant differences are shown in bold.

Abbreviations: add, additive; res, recessive; dom, dominant, over, overdominant; p, level of significance; SNPs, single nucleotide polymorphisms; NF-kB, nuclear factor kappa-light-chain-enhancer of activated B cells; TNFAIP3, TNF alpha induced protein 3; IRAK1, interleukin 1 receptor associated kinase 1; IRAK2, interleukin 1 receptor associated kinase 2; IRAK4, interleukin 1 receptor associated kinase 4; miR, microRNA; MyD88, innate immune signal transduction adaptor; TLR1, toll like receptor 1; TLR4, toll like receptor 4; TNIP1, TNFAIP3 interacting protein 1; TOLLIP, toll interacting protein; TRAF6, TNF receptor associated factor 6.

Supplemental Table S5. Diagnostic accuracy of the predictive models of polymorphisms related to the NF-κB pathway in combination with clinical variables.

	VP	FP	VN	FN	Se	Sp	PPV	NPV
**28 days**								
Clinical variables								
> -1.00 (a)	59	101	14	3	95.2%	12.2%	36.9%	82.4%
> 0.28 (b)	41	21	94	21	66.1%	81.7%	66.1%	81.7%
> 0.79 (c)	21	6	109	41	33.9%	94.8%	77.8%	72.7%
Clinical variables + SNPs								
> -1.31 (a)	59	97	18	3	95.2%	15.7%	37.8%	85.7%
> 0.29 (b)	45	19	96	17	72.6%	83.5%	70.3%	85.0%
> 0.77 (c)	28	5	110	34	45.2%	95.7%	84.8%	76.4%
**90 days**								
Clinical variables								
> -0.65 (a)	93	55	24	5	94.9%	30.4%	62.8%	82.8%
> 0.07 (b)	68	19	60	30	69.4%	75.9%	78.2%	66.7%
> 0.55 (c)	29	4	75	69	29.6%	94.9%	87.9%	52.1%
Clinical variables + SNPs								
> -0.89 (a)	93	59	20	5	94.9%	25.3%	61.2%	80.0%
> -0.21 (b)	77	27	52	21	78.6%	65.8%	74.0%	71.2%
> 0.57 (c)	31	4	75	67	31.6%	94.9%	88.6%	52.8%

Cut-offs: a) Value for 95% of sensitivity; b) Value of sensitivity plus specificity is maximum; c) Value for 95% of specificity.

Abbreviations: FN, false negative; FP, false positive; TN, true negative; TP, true positive; NPV, negative predictive value; PPV, positive predictive value; Se, sensitivity; Sp, specificity. Seven patients were excluded due to missing data for any of the covariates included in the model.
